# The Scheme of the British Medical Association

**Published:** 1918-05-18

**Authors:** C. Thackray Parsons


### A MINISTRY OF HEALTH.

### The Scheme of the British Medical Association.

By C. THACKRAY PARSONS, M.D.Lond.

So long ago as 1868 the British Medical Association urged upon the Government of the day the formation of a Ministry of Health. In January of the present year the Council of the Association approved a scheme for a Ministry of Health, and has now published this scheme, with explanatory notes upon it, in pamphlet form.

The scheme suggests a reorganisation of all the central departments and local authorities dealing with the administration of public health and the provision of medical assistance, with the exception of the medical departments of the Navy, Army, and Air Service, and of the Colonial, India, and Foreign Offices. It recommends that the medical work of the Local Government Board, the Registrar-General's Department, the National Insurance Commissions, the Board of Education, the Home Office, the Privy Council, the Board of Trade, and the Ministry of Pensions should be transferred to a Ministry of Health, consisting of a Minister of Cabinet rank with an Advisory Council. The composition of this Advisory Council is not very clearly defined. It is to be appointed by the Minister "from nominations made by bodies recognised as bearing a special claim to representation," the representatives of the medical profession being nominated by the General Medical Council and the British Medical Association. The Advisory Council is not only to advise the Minister, but is to have the power to report direct to Parliament when its expert opinion is decidedly opposed to action proposed to be taken by the Minister. All or certain of its members are to be paid for actual expenses incurred or services rendered. The staff of the Ministry is to consist of members representing on an equal footing the preventive and clinical sides of medicine, and there is to be a purely scientific department to investigate questions of a scientific nature.

For local administration the scheme suggests the division of the country into suitable areas, being such existing Local Government areas or combinations thereof as would require whole-time administrative medical officers for both clinical and preventive work. These areas might be the counties, county boroughs, or such combination of adjoining non-county boroughs or urban districts as would give a population of 100,000 or 150,000. All matters of health administration within these areas is to be referred to a local Health Committee formed in accordance with a scheme to be sanctioned for each area by the Ministry of Health, and including representatives of the rating authority, of persons contributing to any scheme of National Health Insurance, of the medical profession, and of others engaged in public-health work in the area. If the annual estimate of the expenditure of the local Health Committee involves the raising of a rate of more than 2d. in the pound, the rating authority is to have the right of calling for full information and a conference with the local Health Committee on the matter, and, if no agreement is reached, the disputed matters are to be referred to the Ministry of Health for final decision. In addition, there is to be a statutory Medical Committee directly elected by the medical practitioners in the area. Its duties will be to elect the representatives of the medical profession on the local Health Committee and to advise it on any medical matters, with a right to present its views also to the Ministry of Health and the public.

Each local Health Committee is to have a chief administrative medical officer appointed from any branch of the Service. Under him are to be clinical and preventive staffs, each with its own chief medical officer. The clinical staff will advise on all matters of medical treatment, have administrative control of treatment centres, clinics, and of domiciliary treatment, and co-ordinate this work with that of the special, general, and mental hospitals and infirmaries in the area. The duties of the preventive staff will be those connected with vital statistics and preventive medicine, with the prevention of the spread of infectious disease, and with the investigation of the causes of preventible disease.

The medical staff will consist of whole-time salaried officers engaged in the work of administration and inspection; of institutional medical officers and pathologists, who are to be salaried officers devoting the whole or part of their time to their duties, according to the extent of the work required; and of those engaged in giving domiciliary advice and treatment, who are not to be salaried officers, but are to be paid by some method dependent upon the work done or the number of persons for whose treatment they accept responsibility.

In each area hospitals, clinics, or treatment centres, with a pathological laboratory, are to be recognised or established at which persons entitled to treatment under the public scheme are to be able to obtain institutional, consultative, or specialist services on the recommendation of their medical attendant. Voluntary hospitals are to be encouraged and are to be paid an agreed annual sum by the local Administrative Health Committee in respect of treatment given to patients under the public scheme. Where hospitals wholly maintained by public funds are also required, resident and salaried medical officers are to be appointed, but the main responsibility for treatment is to be in the hands of consulting and visiting practitioners, who, with those at voluntary hospitals treating patients under the public scheme, are to be paid for their services according to the number of patients for whom they accept responsibility. No hospital is to be established in future in any area unless the Ministry of Health is satisfied that it is needed. The scheme suggests the provision of maternity

**A MINISTRY OF HEALTH**—(*continued*).

hospitals for all patients whose home conditions make confinements unusually dangerous or who require operative interference of a major character; of hospitals at which people of moderate means can be received and treated, if they prefer it, by their own family doctor; and of clinical and pathological laboratories, with an effective connection, if possible, with an institution of University rank, doing other work than that for public authorities and associated with research work. All medical students and practitioners are to have reasonable access for educational purposes to the practice at any hospital or institution maintained or aided by public funds. It is strongly urged that there should be effective safeguards against the oppression of public officers for the faithful execution of their duties, with an adequate scale of remuneration for services rendered, and with provision for reasonable superannuation and compensation for disturbance of office.

(*To be continued.*)

